# Analysis of Differentially Expressed Genes in Gastrocnemius Muscle between DGAT1 Transgenic Mice and Wild-Type Mice

**DOI:** 10.1155/2017/5404682

**Published:** 2017-03-13

**Authors:** Fei Ying, Hao Gu, Yuanzhu Xiong, Bo Zuo

**Affiliations:** ^1^Key Laboratory of Swine Genetics and Breeding, Ministry of Agriculture and Key Lab of Agricultural Animal Genetics and Breeding, Ministry of Education, College of Animal Science and Veterinary Medicine, Huazhong Agricultural University, Wuhan 430070, China; ^2^The Cooperative Innovation Center for Sustainable Pig Production, Wuhan 430070, China

## Abstract

Adipose tissue was the major energy deposition site of the mammals and provided the energy for the body and released the external pressure to the internal organs. In animal production, fat deposition in muscle can affect the meat quality, especially the intramuscular fat (IMF) content. Diacylglycerol acyltransferase-1 (DGAT1) was the key enzyme to control the synthesis of the triacylglycerol in adipose tissue. In order to better understand the regulation mechanism of the DGAT1 in the intramuscular fat deposition, the global gene expression profiling was performed in gastrocnemius muscle between DGAT1 transgenic mice and wild-type mice by microarray. 281 differentially expressed transcripts were identified with at least 1.5-fold change and the *p* value < 0.05. 169 transcripts were upregulated and 112 transcripts were downregulated. Ten genes (SREBF1, DUSP1, PLAGL1, FKBP5, ZBTB16, PPP1R3C, CDC14A, GLUL, PDK4, and UCP3) were selected to validate the reliability of the chip's results by the real-time PCR. The finding of RT-PCR was consistent with the gene chip. Seventeen signal pathways were analyzed using KEGG pathway database and the pathways concentrated mainly on the G-protein coupled receptor protein signaling pathway, signal transduction, oxidation-reduction reaction, olfactory receptor activity, protein binding, and zinc ion binding. This study implied a function role of DGAT1 in the synthesis of TAG, insulin resistance, and IMF deposition.

## 1. Introduction

Adipose tissue was the major energy deposited site of the mammals. Also, it provided the energy for the body, kept the temperature stable, and released the external pressure [1, 2]. Adipose tissue can be divided into subcutaneous fat, visceral fat, intermuscular fat, and intramuscular fat by the different deposition site. The intramuscular fat (IMF) was deposited in the muscle tissue [3]. It was the latest formation in adipose tissue. It affected the quality, tenderness, and color of the livestock's production [4–6]. Intramuscular fat was composed of structural fat, phospholipids, and triacylglycerol.

The triacylglycerol (TAG) was the major component of intramuscular fat and it was the important storage molecule of metabolic energy [7, 8]. TAG was one type of neutral lipid, which had a glycerol backbone and three long fatty acids. In animal, the TAG was always in the liver, small intestine, muscle, and adipose tissue. TAG was important for the cell membrane composition and lipoprotein transportation [9]. There were two pathways in the synthesis of TAG [10]. One relied on the acyl-CoA and the other not. The main pathway of TAG synthesis relied on the acyl-CoA [11, 12].

In adipose tissue, the acyl-CoA:diacylglycerol acyltransferase (DGAT) enzyme was the main catalyzer in the last and the only committed step of the major pathway of TAG synthesis [13, 14]. The DGAT enzyme had two isoforms: DGAT1 and DGAT2. DGAT1 was a member of a large family of membrane-bound O-acyltransferases (MBOAT), whereas DGAT2 was a new family [15]. Both the DGAT1 and DGAT2 were the key enzyme in the TAG synthesis, but they had the distinguished function [16, 17]. To determine the biological functions of DGAT1, Chen et al. [18, 19] created the DGAT1-deficient mice. The mice lacking DGAT1 showed significant change in lipid metabolism in several tissues. The DGAT1-deficient mice were resistant to obesity and had increased sensitivity to insulin and leptin. The effects of DGAT1 deficiency on energy and glucose metabolism resulted in part from the altered secretion of adipocyte-derived factors [20, 21]. Otherwise, Buhman et al. [22] used the DGAT1-deficient mice to analyze the triacylglycerol absorption and chylomicron synthesis. They find that DGAT1 is not essential for quantitative dietary triacylglycerol absorption, even in mice fed a high fat diet, or for the synthesis of chylomicrons. Smith et al. [23] demonstrated that DGAT1-deficient mice were viable and can still synthesize triglycerides. The finding indicated that multiple mechanisms exist for triglyceride synthesis. On the contrary, DGAT1 overexpressed in mice increased DGAT1 activity with threefold compared with the WT mice. The overexpression of DGAT1 caused the significant change in fat metabolism, including raised triglyceride synthesis, enhanced fatty acid oxidation, and preserved insulin sensitivity [24, 25].

Research over the past 20 years had predominantly focused on protein coding messenger RNA transcripts and their role in cellular processes, such as disease and development. These whole-transcript array designs provided a complete expression profile of mRNA that impact the mRNA expression profile. Li et al. [26] demonstrated that the overexpression of DGAT1 in the DGAT1 transgenic mice can increase the synthesis of TAG and IMF content, but the mechanism is not clear. In order to better understand and find the possible signal pathway of the DGAT1 regulating the intramuscular fat deposition, we use microarray technology to screen the differentially expressed genes in gastrocnemius muscle between DGAT1 transgenic mice and wild-type mice in this study.

## 2. Materials and Methods 

### 2.1. Plasmid and Transgenic Mice

The construct (MCK-DGAT1) contains, from the 5′-end to the 3′-end, a 7.0 kb porcine MCK promoter, obtained by homologous recombination from porcine bacterial artificial chromosome (GenBank accession number AC139878, Sus scrofa clone RP44-251A2), and a 1.4 kb porcine DGAT1 cDNA without 5′UTR (untranslated region) and 3′UTR, obtained by gene synthesis (GenBank accession number NM_214051.1, Invitrogen, Shanghai, China). TG mice were generated by a standard DNA microinjection [27] of C57BL/6 (Invitrogen, Shanghai, China). Founder TG mice were allowed to mate with wild-type (WT) mice and gave birth to the F1 generation of TG mice (identified by polymerase chain reaction- (PCR-) based genotyping) (see Figure  S1 in the Supplementary Material available online at https://doi.org/10.1155/2017/5404682). Three-month F1 generation male mice gastrocnemius muscles were used in this study. All mice were kept at room temperature (22°C) with a 12 h light/dark cycle. Mice were ad libitum fed a chow diet. All procedures were in accordance with institution guidelines and approved by the Institutional Animal Care and Use Committee of Hubei Province.

### 2.2. Reverse Transcriptase PCR and Real-Time PCR Analysis

The mRNA was extracted from gastrocnemius muscle samples using the RNeasy Lipid Tissue Mini kit (Qiagen, Hilden, Germany) according to manufacturer's protocols. RNA quality was examined using gel electrophoresis (Figure  S2). Total RNA were treated with RNase-free DNase I and subsequently used as a template for first-strand cDNA synthesis using a Revert Aid First Stand cDNA Synthesis Kit (Fermentas Inc., Glen Burnie, MD). Real-time PCR was performed with the SYBR qPCR Mix (Toyobo, Osaka, Japan) in a Bio-Rad CFX96 Real-Time PCR system. The primers sequences were listed in Table 1. The extension time was 30 s. Data were analyzed by the comparative critical threshold method [28], normalized by the amount of *β*-actin mRNA, and expressed relative to the corresponding value in WT mice.

### 2.3. Microarray Experiments

The mRNA was processed for hybridization to Affymetrix mouse gene 2.0 ST arrays (Affymetrix, Santa Clara, CA). These arrays provided whole-transcript coverage, with each of 26,515 genes represented on the array by approximately 27 probes spread across the full length of the gene. A transcript was called detectable if it had more than 7 detected probes. A total of six arrays were used (3 DGAT1 transgenic mice and 3 WT mice), and each array corresponded to labeled RNA from one individual gastrocnemius muscle. The data had submitted to the GEO and the accession number is GSE89192.

### 2.4. Data Analysis

The expression values for each gene were acquired using the GeneChip Operating System (GCOS 1.4, Affymetrix). The expression data from six mice were loaded into Gene-Spring GX 10.0 software (Agilent Technologies) for data normalization and filtering, which were differentially expressed between transgenic animals and control animals. Significance (*p* < 0.05) was calculated using an analysis of variance (ANOVA). Differentially expressed transcripts were identified by cutoff of fold change (FC) ≥ 1.5 and *p* value < 0.05 using unpaired* t*-test.

### 2.5. Bioinformatics Analysis

The gene functions were determined primarily using the NCBI Entrez Gene database. Hierarchical cluster (Ver.2.11) was performed for differentially expressed genes [29]. In order to identify molecular interactions among the genes, Gene Ontology (GO) and pathway-based analysis was carried out on differentially expressed genes to explore if there were significant enrichments of functional categories. Gene Ontology contains three different categories: biological process, cellular components, and molecular function, which were selected to investigate the molecular function of differentially expressed genes. Molecular function of differentially expressed genes was classified according to MAS (molecule annotation system) 3.0 platform (http://bioinfo.capitalbio.com/mas3/). Kyoto Encyclopedia of Genes and Genomes (KEGG) database were used for pathway analysis of differentially expressed genes.

### 2.6. Statistical Analyses

The data were analyzed by least-square analysis of variance procedures using SPSS16.0.0 with fixed effects of genotype and their interaction. We used unpaired 2-tailed Student's* t*-test to evaluate statistical significance. *p* < 0.05 was considered as statistical significant.

## 3. Results and Discussion 

### 3.1. Assessment of the Microarray Quality

We used the PARTEK to analyze the value of the chip signal after homogenization. The picture showed that the signal value distribution of each chip was consistent (Figure  S3). The linear combination of three variables with the highest proportion in the total probe cluster demonstrated that there were significant differences between the transgenic mice (*n* = 3) and the wild-type mice (*n* = 3) (Figure  S4). According to the hierarchical clustering (Figure  S5), we found that three transgenic mice were clustered in one group and three WT mice were in another group.

### 3.2. The Screening of Differentially Expressed Genes

In the gene microarray chip results, 281 differentially expressed transcripts were identified with at least 1.5-fold change and the *p* value < 0.05. In the 281 transcripts, 169 transcripts were upregulated and 112 were downregulated. By checking the RefSeq in the database, we find that 71 transcripts have the annotation (43 were upregulated and 28 were downregulated) (Table  S1).

### 3.3. Validation of Gene Expression Data by Real-Time PCR

**Figure 1:**
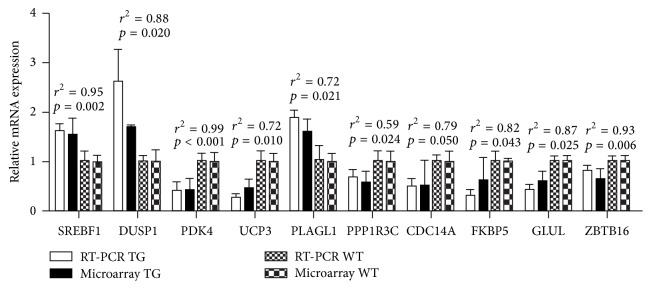
The verification result of microarray by QRT-PCR. The *x*-axis represents the genes; the *y*-axis represents the relative mRNA expression.

To validate the results of the gene chips, RT-PCR was carried out for three upregulated genes (SREBF1, DUSP1, and PLAGL1) and seven downregulated genes (FKBP5, ZBTB16, PPP1R3C, CDC14A, GLUL1, PDK4, and UCP3). As shown in Figure 1, the relative fold differences in the gene expression as determined by the RT-PCR were similar to the results of the microarray analysis. Meanwhile, the *p* value is less than 0.05 and *r*^2^ is more than 0.5.

### 3.4. The Signal Pathway Analysis

**Figure 2:**
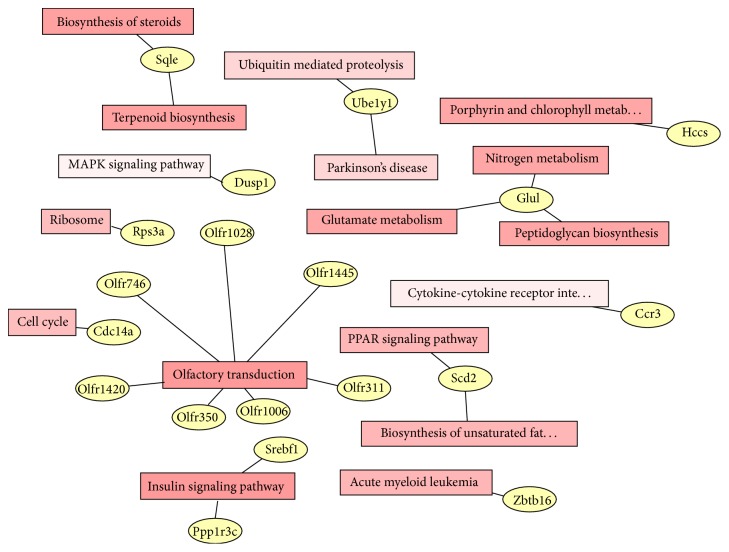
Pathway network of differentially expressed genes. The yellow elliptical box means the differentially expressed genes and the quadrate box means the signal pathway.

To categorize the differentially expressed genes, we used the Kyoto Encyclopedia of Genes and Genomes (KEGG) analysis. It showed that the genes were in lots of signal pathways (Table 2), such as the insulin signaling pathway, the PPAR signaling pathway, and the biosynthesis of unsaturated fatty acids pathway. Otherwise, we find that several important genes simultaneously participated in some biological process and signal pathways by analyzing the network of the signal pathway and genes (Figure 2). As shown in Figure 2, the Glul regulated the glutamate metabolism, the nitrogen metabolism, and peptidoglycan biosynthesis.

### 3.5. The Gene Ontology (G0) Classification

**Figure 3:**
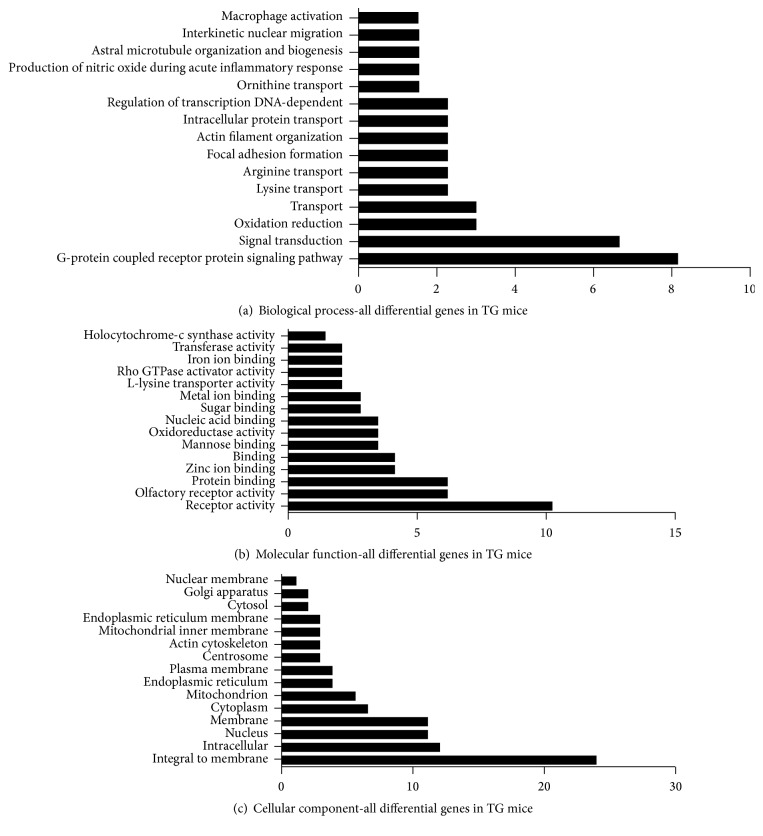
Gene ontology analysis of all differentially expressed genes.

The GO analysis assigned different genes to the different kind of categories. By importing the data into the MAS 3.0 database, all the genes were analyzed together without distinguishing upregulated and downregulated genes. In the results (Figure 3), 78 genes (47.56%) were classified into biological process, 63 genes (38.41%) were in molecular function, and 23 genes (14.02%) were in cellular component.

### 3.6. Interaction Analysis of Differentially Expressed Genes Encoding Proteins

**Figure 4:**
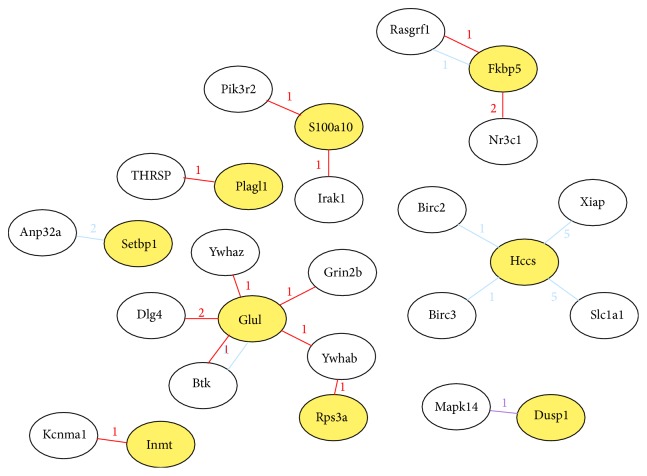
Network of protein interaction. Red line represents InAct database, blue line represents MINT database, and purple line represents DIP database. The number on the line means the subtype of the protein.

We used four databases (MINT, HPRD, InAct, and DIP) supported by the MAS 3.0 to analyze the interaction between the proteins which were the differentially expressed genes translated (Figure 4). The Fkbp5 protein interacted with 2 subtypes of the Nr3c1 protein.

### 3.7. Detection of the Global Gene Expression in the Gastrocnemius of the DGAT1 Transgenic Mice and WT Mice by Microarray

In our study, the gene expression differences were processed by the Affymetrix mouse gene 2.0 st. Microarray data revealed that many genes were more than 1.5-fold change and *p* < 0.05 (43 were upregulated and 28 were downregulated). Ten genes (SREBF1, DUSP1, PLAGL1, FKBP5, ZBTB16, PPP1R3C, CDC14A, GLUL, PDK4, and UCP3) were selected for validation by RT-PCR. All of them show the significant differential expression level. Furthermore, the trends were same between the results of two methods, showing the reliability of the microarray analysis. It is reasonable to expect that these genes were associated with the DGAT function.

The GO analysis demonstrated that three major kinds of categories are connected with the differentially expressed genes, including G-protein coupled receptor protein signal pathway, receptor activity, and integral membrane. Meanwhile, the KEGG pathway showed that some of genes were involved in the insulin signal pathway (SREBF1, PDK4, and PPP1R3C), the MAPK signaling pathway (DUSP1), and the biosynthesis of unsaturated fatty acids pathway (SCD2, UCP3).

### 3.8. The Differentially Expressed Genes Associated with the Glucose Metabolism

Pyruvate dehydrogenase complex (PDC) was a key enzyme in the process of tricarboxylic acid cycle, which can promote the decarboxylation of the acetyl CoA [30]. However, it was inhibited by pyruvate dehydrogenase kinase (PDK). PDK4 was a subtype of the PDK family. PDK4 can regulate the synthesis of glycine and the production of glycolysis [31]. Wang et al. discovered that knocking out the PDK4 could decrease the level of blood sugar and improve the tolerability of the glucose. In our study, the PDK4 in the DGAT1-overexpression mice was decreased by 2.4-fold. This indicated that the overexpression of DGAT1 could relieve the inhibition of PDC by downregulating the PDK4 and then promote the process of TAG synthesis. At last, the glucose utilization was increased and insulin resistance was avoided.

PP1 family was a phosphoric acid protease family. It can regulate glycogen synthetase (GS) and glycogen phosphorylase (GP) activity and control the synthesis and decomposition of the glycogen [32]. As a member of the PP1 family, PPP1R3C was expressed mainly in the muscle and liver. PPP1R3C participated in glucose metabolism and glycogen synthesis. The high glucose stimulated its expression [33]. In our experiment, the PPP1R3C was 1.7-fold downregulated. So we inferred that DGAT1 regulated the insulin sensitivity and the metabolism of glucose by the PPP1R3C.

### 3.9. The Differentially Expressed Genes Associated with the Lipogenesis

Sterol regulatory element binding protein (SREBF1) is a membrane binding protein. It can be in the role of proteases shuttle in the nucleus and induce the lipogenesis gene expression [34]. SREBF1 can regulate the synthesis of the fatty acid and sterols. Also, it was the key factor of the TAG metabolism and can regulate the lipid metabolism by the insulin signaling pathway [35]. The upexpression of SREBF1 in goat mammary epithelial cells can increase DGAT1 and LPIN1, promoting the synthesis of fatty acids and the deposition of TAG [36]. In the microarray data, the mRNA level of the SREBF1 increased more than 1.5-fold in transgenic mice, revealing that the overexpression of DGAT1 can induce the SREBF1. Furthermore, the DGAT1 increased the content of TAG, affected the sensibility of insulin, and adjusted the body's energy metabolism by the SREBF1. Further studies will be needed to test the protein level of the SREBF1 and find the mechanism between the DGAT1 and SREBF1.

Stearoyl-coenzyme A desaturase (SCD) was a key enzyme that catalyzed the conversion of saturated fatty acids to unsaturated fatty acids, which can regulate the percentage of saturated fatty acids and unsaturated fatty acids in cells. It had 4 subtypes (SCD1, SCD2, SCD3, and SCD4) in the mouse. SCD2 affected the body's energy metabolism by regulating the synthesis of fatty acid and glycolysis through the PPAR signaling pathway. PPAR, as a key factor of lipid synthesis, increased the expression of adiponectin and TAG related genes to regulate glucose utilization and insulin sensitivity [37]. In our study, the expression of SCD 2 was upregulated by 1.7-fold in transgenic mice. SCD1 also increased more than 2-fold, but it was not significant. This change demonstrated that DGAT1 facilitate the formation of fat and energy metabolism by regulating the genes in the PPAR signaling pathway.

### 3.10. The Differentially Expressed Genes Associated with the Lipid Oxidation

Dual specificity phosphatase 1 (DUSP1) was the member of the MKP phosphatase family. It regulated the MAPK signaling pathway by the dephosphorylation. DUSP1-deficient mice increased energy output to avoid the obesity caused by the high fat diet [38, 39]. DUSP1 can directly inhibit PGC-1 alpha by the p38-MAPK pathway. Furthermore, DUSP1 changed the muscle fiber type by PGC-1 alpha, thus affecting the composition of muscle fiber [40]. DUSP1 can also affect the metabolism of fatty acids. Knocking out DUSP1 can enhance the oxidation of fatty acids and control the body's energy output [41]. In our data, the expression of DUSP1 increased 1.7-fold in transgenic mice, indicating the overexpression of DGAT1 can upregulate DUSP1. And it can promote the dephosphorylation of the p38-MAPK and inhibit the synthesis of mitochondria. In particular, the upregulation of DUSP1 can enhance the expansion of type I muscle fiber by PGC-1*α*. As we know, the type I muscle fiber had a higher content of TAG and glucose transporter 4 (GLUT4). So, the overexpression of DGAT1 regulated the synthesis of TAG and energy metabolism by the MAPK signaling pathway and PGC-1*α*. Therefore, we think that DGAT1 controlled the deposition of fat by the DUSP1.

Uncoupling protein 3 (UCP3) belonged to the mitochondrial carrier protein family and was mainly expressed in skeletal muscle and brown adipose tissue. UCP3 was mainly involved in the uncoupling effect of mitochondrial respiratory chain and played an important role in the maintenance of mitochondrial activity, the adaptability of the body thermogenesis, and the fatty acid oxidation [42, 43]. The overexpression of UCP3 increased the feed intake and reduced blood glucose and insulin levels in mice [44]. Accumulating the more fatty acids in the liver cells induced UCP3 expression. UCP3 activated fatty acid metabolism genes, changed the permeability of the mitochondrial inner membrane, and reduced the damage of liver cell. In the study, UCP3 decreased more than 2.0-fold. This result showed that the energy output of DGAT1 transgenic mice was relatively low, and the fat deposition was faster. This was consistent with the bigger body size of DGAT1 transgenic mice as discovered previously, comparing with the WT mice. So the DGAT1 can increase the level of insulin in the blood and the body weight and fat by the UCP3.

Zinc finger and BTB domain containing (ZBTB) 16 was a transcriptional inhibitor, belonging to the proteins' superfamily. The expression levels of ZBTB16 in brown adipose tissue and skeletal muscle were 15.3 and 2.1 times higher in cold-stimulated mice, respectively. The overexpression of ZBTB16 increased the number of mitochondria and the glucose consumption and reduced the content of the TAG in the cell. In our study, the mRNA level of ZBTB16 decreased 1.5-fold. It revealed that DGAT1 can reduce the oxidation of fatty acids and the energy output of the body by the ZBTB16, which were better for the deposition of intramuscular fat.

Taken together, glucose and lipid metabolism was regulated and the reaction of the TAG synthesis was catalyzed by the overexpression of DGAT1. Afterwards, the TAG synthesis was sufficient to improve muscle insulin sensitivity and increase the content of IMF. As a result, the upregulation of DGAT1 led to insulin sensitivity and IMF improvement.

## 4. Conclusion

In our study, we used the microarray to detect the differential gene expression in the three-month DGAT1 transgenic mice and WT mice. Furthermore, we found 7 differentially expressed genes involved in glucose metabolism, lipogenesis, and lipid oxidation, which were found to be connected with IMF content. As the previous evidences show, SREBF1, UCP3, and SCD2 participated in the DGAT1 regulation, which was consistent with our results. Moreover, there were no reports about the regulation of DUSP1, PPP1R3C, PDK4, and ZBTB16 by DGAT1 until now. So these genes identified in our study merit further investigation to understand the signal pathway of the DGAT1 regulating the synthesis of TAG and the formation of IMF. Thus, we inferred that DGAT1 regulates the synthesis of TAG and the formation of IMF by possibly affecting the insulin signaling pathway, the MAPK signaling pathway, the biosynthesis of unsaturated fatty acids, and so on. This research may open a possible means to inhibit insulin resistance and treat type 2 diabetes.

## Supplementary Material


**Fig** **S** **1** Identified by polymerase chain reaction(PCR)-based genotyping F1 generation of TG mice. M maker DL2000 1,2,5 transgenic mice, 3,4,6 WT mice, N negative control.
**Fig** **S** **2** The result of RNA gel electrophoresis. C1,C2,C3 WT mice, C4,C5,C6 transgenic mice.
**Fig** **S** **3** The result of chip signal value distribution. The X-axis represents the signal of the probe, the Y-axis represents the number of probe. The different color is the different sample(red, deep blue and light blue are transgenic mice; green, purple, orange are WT mice). 1-28-1, 1-28-5 and 1601-1-1 are the transgenic mice; 3YCY1, 3YCY2, 3YCY3 are the wild-type mice.
**Fig** **S** **4** The result of principal component analysis. The different color of the ball is the different sample (red, deep blue and light blue are transgenic mice; green, purple, orange are WT mice). The purple cycle is transgenic group and the yellow cycle is WT group. 1-28-1, 1-28-5 and 1601-1-1 are the transgenic mice; 3YCY1, 3YCY2, 3YCY3 are the wild-type mice.
**Fig** **S** **5** Hierarchical cluster result of differential expressed genes. The X-axis represents the probe, the Y-axis represents the sample(red, orange, deep blue are transgenic mice and yellow, green, light blue are WT mice). The different of the color means the distance of the cluster. 1-28-1, 1-28-5 and 1601-1-1 are the transgenic mice; 3YCY1, 3YCY2, 3YCY3 are the wild-type mice.
Table S1 43 up-regulated and 28 down-regulated genes in the microarray analysis.

## Figures and Tables

**Table 1:** Primer information of differentially expressed genes used for real-time PCR.

Gene name	Primer sequences (5′-3′)	Tm (°C)	Product size
SREBF1	CAGAACTGGGACCTTGGGAC CCCAGCTCCTCTGTCTTTGG	60	162
UCP3	CTGCACCGCCAGATGAGTTT ATCATGGCTTGAAATCGGACC	60	191
DUSP1	AGTGCCTATCACGCTTCTCG GGAGCTGATGTCTGCCTTGT	58	153
FKBP5	CTGCACCGCCAGATGAGTTT AAAGAAAAGCTGACGCAGGC	60	140
ZBTB16	CTGGGACTTTGTGCGATGTG CGGTGGAAGAGGATCTCAAACA	60	106
PDK4	AGGGAGGTCGAGCTGTTCTC GGAGTGTTCACTAAGCGGTCA	60	185
GLUL	CAACGACTTTTCTGCCGGTG TATTGGAAGGGTTCGTCGCC	59	186
PLAGL1	CACCTCACTCGTCACACCAA TGAAGGCGCAATGAGTTGGA	60	102
CDC14A	GATAACATCGTGCGGAGATTCC CATAACAGGCTATCAATGTCCCG	60	109
PPP1R3C	TGCAATGGAAACCTGACGGA AAGTTCTCCACTCTCCCCCA	60	163
*β*-Actin	AGGCCCAGAGCAAGAGAGGTA GGGGTGTTGAAGGTCTCAAACA	60	220

**Table 2:** The pathway information of some differentially expressed genes.

Pathway	Gene symbol	Gene title
Olfactory transduction	Olfr1445 Olfr350 Olfr746 Olfr311 Olfr1028 Olfr1420 Olfr1006	Olfactory receptor 1445 Olfactory receptor 350 Olfactory receptor 746 Olfactory receptor 311 Olfactory receptor 1028 Olfactory receptor 1420 Olfactory receptor 1006

Insulin signaling pathway	Srebf1	Sterol regulatory element binding transcription factor 1
	Ppp1r3c	Protein phosphatase 1, regulatory (inhibitor) subunit

Peptidoglycan biosynthesis	Glul	Glutamate-ammonia ligase

Terpenoid biosynthesis	Sqle	Squalene epoxidase

Nitrogen metabolism	Glul	Glutamate-ammonia ligase

Biosynthesis of steroids	Sqle	Squalene epoxidase

Biosynthesis of unsaturated fatty acids	Scd2	Stearoyl-coenzyme A desaturase 2

Glutamate metabolism	Glul	Glutamate-ammonia ligase

Porphyrin and chlorophyll metabolism	Hccs	Holocytochrome-c synthetase

Acute myeloid leukemia	Zbtb16	Zinc finger and BTB domain containing 16

PPAR signaling pathway	Scd2	Stearoyl-coenzyme A desaturase 2

Ribosome	Rps3a	Ribosomal protein S3A

Cell cycle	Cdc14a	CDC14 cell division cycle 14A

Ubiquitin mediated proteolysis	Ube1y1	Ubiquitin-activating enzyme E1, Chr Y 1

Parkinson's disease	Ube1y1	Ubiquitin-activating enzyme E1, Chr Y 1

Cytokine-cytokine receptor interaction	Ccr3	Chemokine (C-C motif) receptor 3

MAPK signaling pathway	Dusp1	Dual specificity phosphatase 1
