# Supplementary material for: Analysis of Differentially Expressed Genes in Gastrocnemius Muscle between DGAT1 Transgenic Mice and Wild-Type Mice
Source: Biomed Res Int. 2017 Mar 13;2017:5404682. doi: 10.1155/2017/5404682 (PMC5366756; doi:10.1155/2017/5404682)
Supplement: Supplementary file 1 — Fig S 1 Identified by polymerase chain reaction(PCR)-based genotyping F1 generation of TG mice. M maker DL2000 1,2,5 transgenic mice, 3,4,6 WT mice, N negative control. Fig S 2 The result of RNA gel electrophoresis. C1,C2,C3 WT mice, C4,C5,C6 transgenic mice. Fig S 3 The result of chip signal value distribution. The X-axis represents the signal of the probe, the Y-axis represents the number of probe. The different color is the different sample(red, deep blue and light blue are transgenic mice; green, purple, orange are WT mice). 1-28-1, 1-28-5 and 1601-1-1 are the transgenic mice; 3YCY1, 3YCY2, 3YCY3 are the wild-type mice. Fig S 4 The result of principal component analysis. The different color of the ball is the different sample (red, deep blue and light blue are transgenic mice; green, purple, orange are WT mice). The purple cycle is transgenic group and the yellow cycle is WT group. 1-28-1, 1-28-5 and 1601-1-1 are the transgenic mice; 3YCY1, 3YCY2, 3YCY3 are the wild-type mice. Fig S 5 Hierarchical cluster result of differential expressed genes. The X-axis represents the probe, the Y-axis represents the sample(red, orange, deep blue are transgenic mice and yellow, green, light blue are WT mice). The different of the color means the distance of the cluster. 1-28-1, 1-28-5 and 1601-1-1 are the transgenic mice; 3YCY1, 3YCY2, 3YCY3 are the wild-type mice. Table S1 43 up-regulated and 28 down-regulated genes in the microarray analysis. [file 5404682.f1.docx]

**
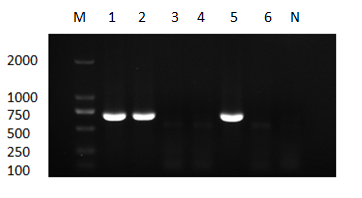
**

**Fig S1** Identified by polymerase chain reaction(PCR)-based genotyping F1 generation of TG mice. M maker DL2000 1,2,5 transgenic mice, 3,4,6 WT mice, N negative control.


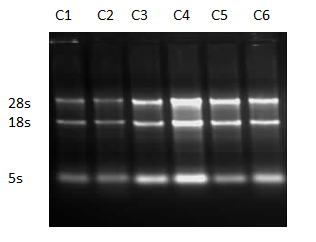


**Fig S2** The result of RNA gel electrophoresis. C1,C2,C3 WT mice, C4,C5,C6 transgenic mice.


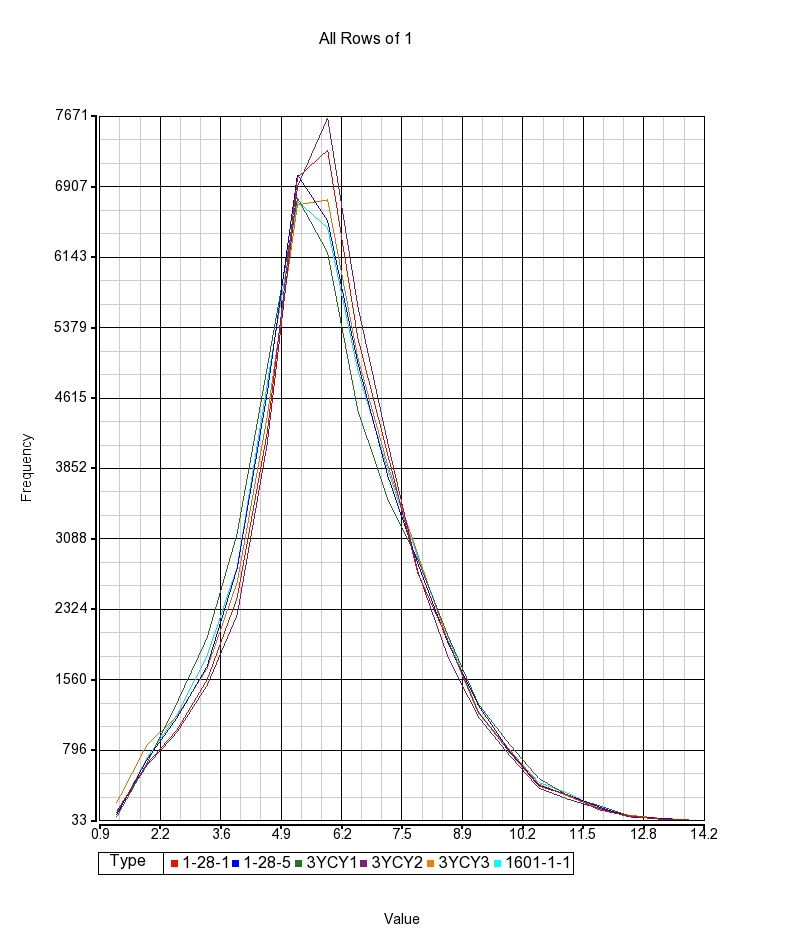


**Fig S3** The result of chip signal value distribution. The X-axis represents the signal of the probe, the Y-axis represents the number of probe. The different color is the different sample(red, deep blue and light blue are transgenic mice; green, purple, orange are WT mice). 1-28-1, 1-28-5 and 1601-1-1 are the transgenic mice; 3YCY1, 3YCY2, 3YCY3 are the wild-type mice.


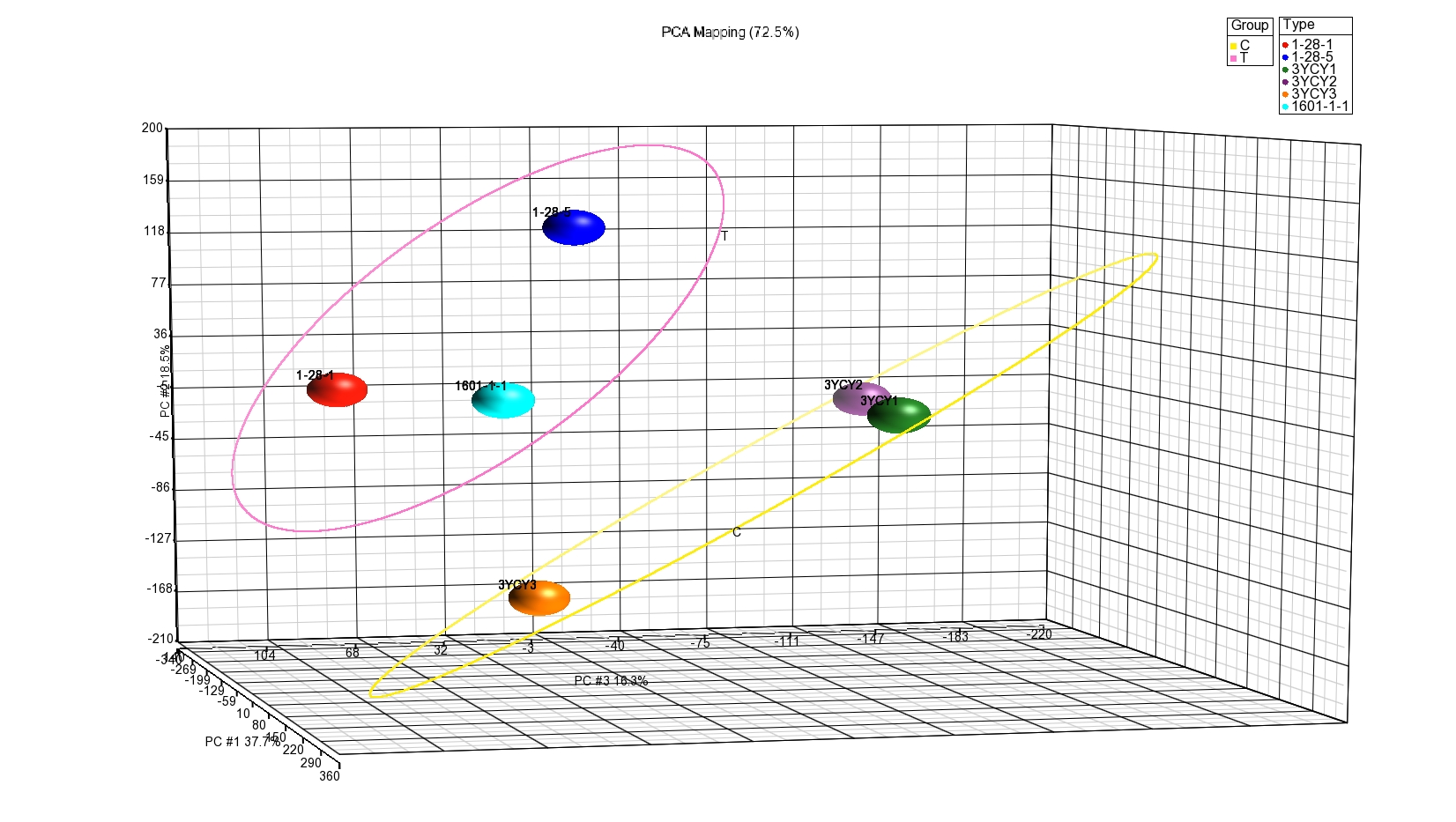


**Fig S4** The result of principal component analysis. The different color of the ball is the different sample (red, deep blue and light blue are transgenic mice; green, purple, orange are WT mice). The purple cycle is transgenic group and the yellow cycle is WT group. 1-28-1, 1-28-5 and 1601-1-1 are the transgenic mice; 3YCY1, 3YCY2, 3YCY3 are the wild-type mice.


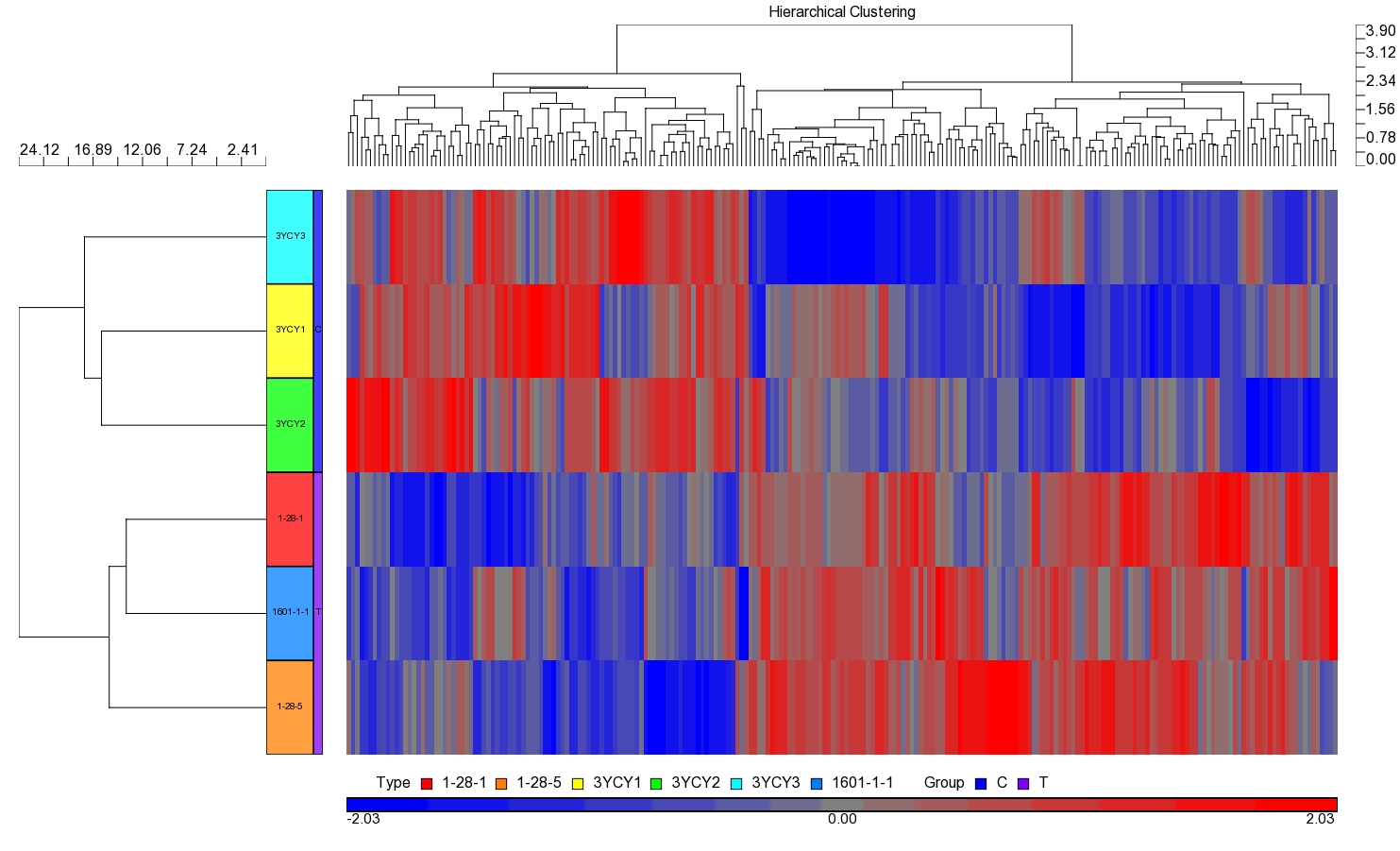


**Fig S5** Hierarchical cluster result of differential expressed genes. The X-axis represents the probe, the Y-axis represents the sample(red, orange, deep blue are transgenic mice and yellow, green, light blue are WT mice). The different of the color means the distance of the cluster. 1-28-1, 1-28-5 and 1601-1-1 are the transgenic mice; 3YCY1, 3YCY2, 3YCY3 are the wild-type mice.

Table S1 43 up-regulated and 28 down-regulated genes in the microarray analysis

| **Gene description (Symbol)** | **RefSeq** | **P-value** | **Fold change** |
| --- | --- | --- | --- |
| Immunoglobulin kappa chain variable 1  (Igk-V1) | Z95477 | 0.034007 | 3.22843 |
| Interferon activated gene 202B  (Ifi202b) | NM_008327 | 0.033115 | 2.42952 |
| Aly/REF export factor pseudogene  (Gm6489) | ENSMUST00000152491 | 0.037128 | 2.03965 |
| MicroRNA 3473b  (Mir3473b) | NR_039554 | 0.032037 | 1.97909 |
| CD209d antigen  (Cd209d) | ENSMUST00000011445 | 0.005603 | 1.95782 |
| Membrane-spanning 4-domains, subfamily A  (Ms4a4c) | NM_029499 | 0.003145 | 1.80668 |
| Olfactory receptor 1445  (Olfr1445) | ENSMUST00000049624 | 0.026491 | 1.79976 |
| Olfactory receptor 350  (Olfr350) | NM_146627 | 0.049587 | 1.76118 |
| Chemokine (C-C motif) receptor 3  (Ccr3) | NM_009914 | 0.002521 | 1.75132 |
| Stearoyl-Coenzyme A desaturase 2  (Scd2) | NM_009128 | 0.000119 | 1.74597 |
| Y1 small cytoplasmic  (Rny1) | NR_004419 | 0.025757 | 1.72017 |
| Spermatogenesis associated glutamate (E)-rich  (Speer4f) | NM_027609 | 0.019211 | 1.71896 |
| Vomeronasal 2, receptor 117 (Vmn2r117) | AB540948 | 0.047101 | 1.71675 |
| LOC100862171 | XR_141817 | 0.040465 | 1.71595 |
| Olfactory receptor 1182 (Olfr1182) | NM_001011535 | 0.044757 | 1.70417 |
| Olfactory receptor 746 (Olfr746) | NM_146298 | 0.0058 | 1.7025 |
| Dual specificity phosphatase 1 (Dusp1) | ENSMUST00000025025 | 0.021805 | 1.70103 |
| RIKEN cDNA 1700010M22 gene  (1700010M22Rik) | NM_025490 | 0.029418 | 1.69343 |
| Male-specific lethal 3 homolog (Msl3) | NM_010832 | 0.00734 | 1.69073 |
| Olfactory receptor 311 (Olfr311) | NM_146537 | 0.008721 | 1.68333 |
| Rho GTPase activating protein 6  (Arhgap6) | NM_009707 | 0.027267 | 1.67905 |
| Holocytochrome c synthetase (Hccs) | NM_008222 | 0.013753 | 1.6712 |
| CD209f antigen  (Cd209f) | ENSMUST00000145007 | 0.003569 | 1.66165 |
| Olfactory receptor 1028 (Olfr1028) | NM_001011774 | 0.015417 | 1.63059 |
| Predicted gene 454  (Gm454) | AK138922 | 0.036782 | 1.619 |
| T cell receptor alpha variable 12-2  (Trav12-2) | X04330 | 0.004905 | 1.61475 |
| S100 calcium binding protein A10  (S100a10) | NM_009112 | 0.02488 | 1.6128 |
| Predicted gene 14322 (Gm14322) | NM_001243903 | 0.00095 | 1.60874 |
| Vomeronasal 1 receptor 23 (Vmn1r23) | NM_134179 | 0.034641 | 1.60505 |
| Pleiomorphic adenoma gene-like 1  (Plagl1) | NM_009538 | 0.019248 | 1.60416 |
| Squalene epoxidase  (Sqle) | NM_009270 | 0.018043 | 1.60247 |
| Ribosomal protein S3A (Rps3a) | BC084675 | 0.010619 | 1.58546 |
| Vomeronasal 1 receptor 21 (Vmn1r21) | ENSMUST00000081186 | 0.001702 | 1.5725 |
| Cytochrome P450, family 4, subfamily a  (Cyp4a32) | NM_001100181 | 0.037971 | 1.57198 |
| Predicted gene 3716 (Gm3716) | NR_045078 | 0.02254 | 1.56901 |
| Olfactory receptor 191 (Olfr191) | NM_001011807 | 0.033769 | 1.56502 |
| MicroRNA 3962  (Mir3962) | NR_039539 | 0.026645 | 1.55046 |
| Sterol regulatory element binding transcription factor 1 (Srebf1) | NM_011480 | 0.021955 | 1.53975 |
|  |  |  |  |
| Predicted gene 15032  (Gm15032) | XM_003086859 | 0.027926 | 1.53747 |
| Predicted gene 11546  (Gm11546) | XM_001474160 | 0.042783 | 1.52853 |
| Family with sequence similarity 177, member A  (Fam177a) | BC048158 | 0.038732 | 1.52436 |
| Predicted gene, 19333  (Gm19333) | XR_105044 | 0.031635 | 1.51859 |
| Olfactory receptor 1420  (Olfr1420) | ENSMUST00000072784 | 0.021066 | 1.50559 |
| Outer dense fiber of sperm tails 3-like 2  (Odf3l2) | NM_001033473 | 0.016467 | 0.416239 |
| Pyruvate dehydrogenase kinase, isoenzyme 4  (Pdk4) | NM_013743 | 0.033899 | 0.420358 |
| Uncoupling protein 3  (Ucp3) | NM_009464 | 0.025803 | 0.454573 |
| RIKEN cDNA 3110070M22 gene  (3110070M22Rik) | NR_027974 | 0.007807 | 0.466375 |
| Indolethylamine N-methyltransferase  (Inmt) | ENSMUST00000003569 | 0.015562 | 0.507473 |
| Olfactory receptor 1006 (Olfr1006) | NM_146570 | 0.010229 | 0.508956 |
| CDC14 cell division cycle 14A (Cdc14a) | NM_001080818 | 0.011438 | 0.516543 |
| Predicted gene 4861 (Gm4861) | NM_177665 | 0.017856 | 0.52283 |
| Ubiquitin-activating enzyme E1, Chr Y 1  (Ube1y1) | NM_011667 | 0.03455 | 0.537835 |
| MicroRNA 3098  (Mir3098) | NR_037281 | 0.042555 | 0.554697 |
| Zinc finger protein 457 (Zfp457) | NM_001003666 | 0.001792 | 0.568476 |
| L antigen family member 3-like (LOC100503280) | NM_001251890 | 0.002177 | 0.571143 |
| Solute carrier family 7  (Slc7a2) | ENSMUST00000057784 | 0.040433 | 0.572784 |
| Protein phosphatase 1, regulatory  (Ppp1r3c) | ENSMUST00000087321 | 0.022577 | 0.579943 |
| MicroRNA let7c-2  (Mirlet7c-2) | NR_029729 | 0.041782 | 0.587988 |
| Glutamate-ammonia ligase (Glul) | NM_008131 | 0.039429 | 0.593225 |
| Transforming, acidic coiled-coil containing protein (Tacc2) | NM_001004468 | 0.000493 | 0.598986 |
| FK506 binding protein 5 (Fkbp5) | ENSMUST00000114792 | 0.017089 | 0.614112 |
| ADP-ribosylation factor interacting protein  (Arfip1) | ENSMUST00000154148 | 0.040025 | 0.630457 |
| REV3-like, catalytic subunit of DNA polymerase zeta  (Rev3l) | NM_011264 | 0.008113 | 0.632246 |
| Zinc finger and BTB domain containing 16  (Zbtb16) | NM_001033324 | 0.022525 | 0.635084 |
| Perilipin 4  (Plin4) | NM_020568 | 0.008229 | 0.635938 |
| RIKEN cDNA 6430548M08 gene  (6430548M08Rik) | NM_001163760 | 0.001061 | 0.646756 |
| SET binding protein 1  (Setbp1) | NM_053099 | 0.040923 | 0.651501 |
| Predicted gene 10499  (Gm10499) | XM_003085130 | 0.007464 | 0.659705 |
| Predicted gene 514  (Gm514) | NM_001111145 | 0.005758 | 0.660184 |
| Predicted gene 11563  (Gm11563) | NM_001126320 | 0.039716 | 0.661284 |
| RIKEN cDNA 0610038B21 gene  (0610038B21Rik) | NR_028125 | 0.037744 | 0.665945 |
